# Design of an Automated Multiposition Dynamic Wheelchair

**DOI:** 10.3390/s21227533

**Published:** 2021-11-12

**Authors:** Luis Antonio Aguilar-Pérez, Juan Carlos Paredes-Rojas, Jose Israel Sanchez-Cruz, Jose Alfredo Leal-Naranjo, Armando Oropeza-Osornio, Christopher Rene Torres-SanMiguel

**Affiliations:** 1 Instituto Politécnico Nacional, Escuela Superior de Ingeniería Mecánica y Eléctrica Unidad Zacatenco, Sección de Estudios de Posgrado e Investigación Unidad Zacatenco, Ciudad de México 07738, Mexico; laguilarp@ipn.mx (L.A.A.-P.); jsanchezc1107@alumno.ipn.mx (J.I.S.-C.); 2 Instituto Politécnico Nacional, Centro Mexicano para la Producción más Limpia, Acueducto de Guadalupe S/N, La laguna Ticomán, Ciudad de México 07340, Mexico; jparedes@ipn.mx; 3 School of Engineering, University of Liverpool, Brownlow Hill, Liverpool L69 3GH, UK; jose.leal@liverpool.ac.uk; 4 Instituto Politécnico Nacional, Escuela Superior de Ingeniería Mecánica y Eléctrica, Unidad Ticomán, Ciudad de México 07340, Mexico; aoropeza@ipn.mx

**Keywords:** biomechanics, wheelchair design, service robots

## Abstract

This work presents a design for an automatized multiposition dynamic wheelchair used to transport quadriplegic patients by reconfiguring a manual wheelchair structure. An electric actuator is attached to a four-bar mechanism fixed to each side of a wheelchair’s backrest to reach multiposition. The entire device is actuated through a PID controller. An experimental test is carried out in a simplified wheelchair structure. Finally, the structure of the wheelchair is evaluated through the Dynamic analysis and Finite Element Method under the payload computed with the most critical position reached by the mechanism.

## 1. Introduction

In recent years Research Institutes around the world have been looking to increase the quality of life of spinal cord injury (SCI) people by the development of different devices like assistive robots, the design of a mechanism for therapies and devices to transport patients between different zones of the hospital or house [1,2,3,4,5,6,7,8]. There are several examples of devices [9,10,11,12,13,14,15,16,17]. Alkhateeb shows one of them, where the seat’s effect to back support angle adjustments on head, neck, and shoulder postural alignment in people with cerebral palsy [18]. The work presented by Hong determined if people with several types of wheelchair backrests experience distinct comfort levels. The higher discomfort ratings among rigid backrest users with tetraplegia may be due to sub-optimal shape, fit, or adjustment [19].

In the same way, Murray showed an innovative work of healthy posture wheelchair movement control. This work presents a design to help users achieve a range of functional tasks without fear of falling and encourage a healthy posture [20]. Also, Shirogane has shown that the force applied on the foot support accounted for 70–80% of the total load in wheelchair structures [21]. Other studies have reported an optimized positioning and posture of customized postural support when standard rigid back support is incongruent with a person’s back contours. There are wheelchairs completely designed for SCI people where the purpose is to develop back supports that would enhance postural support for those with spinal deformities [22]. Quadriplegic people need constant postural changes to reduce skin injuries like an ulcer. Those injuries arise due to skin contact with rough surfaces, inadequate vents, and a lengthy period in the same position. Even using soft materials, which help redistribute the pressure applied over the skin, the postural pressure still produces injuries. Most of the designs are only used to adopt one fixed position and transport the patient or assume a lay-down position. Those imply the use of two different devices to satisfy both necessities.

The postural position of disabled people was demonstrated to change the center of gravity’s balance and decrease the pressure applied over the skin. With the previous idea, the present work shows the design of a wheelchair that achieves various positions by using only a four-bar mechanism attached to the mainframe of the wheelchair. One of the proposed goals is to use a standard wheelchair and modify its structure. The proposed methodology is divided into four steps. The first uses the analytical tool of the Inventive Problem-Solving Theory (TRIZ) to a design solution. Second, a dynamical simulation of the four-bar mechanism is computed using the dimensional and functional parameters of the proposed design. Third, finite Element Analysis of the leading wheelchair components is done under static conditions. Finally, a PID controller is planned by using multibody simulation for the full results. The main contribution of this work consists of the mechanical design and control of a wheelchair that is capable of taking multiple positions until it becomes a stretcher. The movement is generated by a four bars mechanism that quickly balances the resistance and stability that the wheelchair requires to ensure an optimal position for the occupant. One fact to consider regarding the design of this multiposition wheelchair is that it may facilitate the disabled patient to be transferred to the bed, since when the chair takes the position of a stretcher, the height of this is similar to a standard bed, and the armrest can be removed.

## 2. Backgrounds and Design Requirements

**Figure 1:**
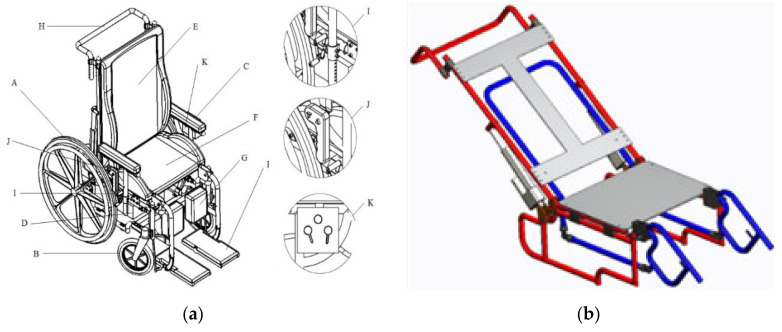
Wheelchair CAD model; (**a**) Wheelchair components, (**b**) Support and mechanism parts of the wheelchair structure.

The multiposition wheelchair components are shown in Figure 1a and Table 1. The design is composed of 371 pieces and considers all the necessary elements to carry out multiple positions. Figure 1b shows the support structures in red color and blue color’s footrests. The four-bar mechanism is used to reconfigure the backrest position.

Concerning the overall design of the wheelchair, it is done with ergonomics encompassing the seat base, footrest, backrest, and all parts that integrate the wheelchair. The four-bar mechanism is driven by an electromechanical actuator fixed to the backrest of the wheelchair, and it is controlled by using a PIC16F887 which oversees processing the system’s information. Table 2 summarizes the principal characteristics needed to design a wheelchair with a postural reconfiguration position.

**Figure 2:**
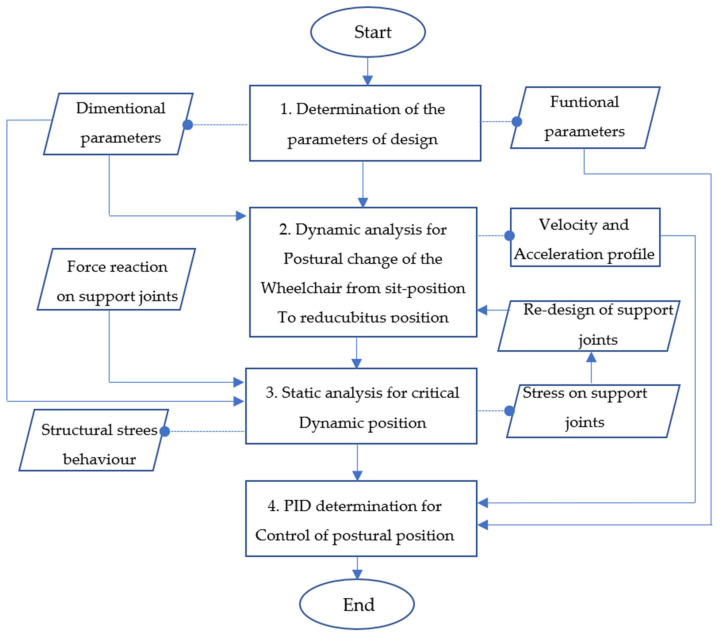
Wheelchair mechanism methodology.

The proposed methodology used to achieve this design is summarized in Figure 2. The structured steps are shown in the block diagram establish the logical sequence for the design and analysis of the wheelchair with postural changes. This structure was proposed through principles, processes, and stages to elaborate the design of the mechanism applied to the postural automation of the wheelchair.

The first step (Identification of the parameters of design) refers to finding the functional and dimensional parameters of the design. The substance field analysis or Su-Field analysis is the Inventive Problem Solving (TRIZ), an analytical tool used to model problems related to existing systems [23]. This methodology is based on the following definition: by describing a specific problem into a generic one, it is possible to seek generic solutions and infer a specific solution from the generic suggestions. The second step (Dynamic analysis of postural change from sit-position to decubitus position) of the proposed methodology proposes to compute the dynamics response of the mechanism to evaluate the payload applied on the gluteus for a postural change of the wheelchair from a sit position to a decubitus position. Thus, the computed results allow us to determine the force reaction applied over the joints of the backrest support and, in case of necessity, to reshape to support the dynamic loads. The third step evaluates the stress of the structure under a static critical condition. The result of this step (Statics analysis for critical dynamic position) gives information about the structure behavior itself. In the last step (PID determination for control of postural position), a digital PID controller is computed to guarantee that any position can be reached under dynamical and stress values.

## 3. The Substance Field Analysis

Almost any patient with decreased mobility (considered a quadriplegic patient) inevitably has an ulcer on their back and gluteus. These injuries are caused by different effects when the patient remains in the same position for extended periods. One of them is the dropsy disease. Dropsy refers to the uncontrolled swelling of soft tissues due to the accumulation of excess water. This problem causes an increment in the weight of the limbs and overloads the body areas previously mentioned.

Another problem is the loss of corporal mass that causes their hip bones to apply concentrated loads in the patient’s back and gluteus. Also, the static position in certain areas increases the temperature of the body regions in contact with the bed or the wheelchair used to transport or rest the patient. This increase in the temperature causes the skin to perspire, which is also related to the increased humidity needed to maintain the skin’s optimal conditions. Thus, ulcer formation is related to a rupture of the skin tissue. This rupture happens due to the layers of the skin decreasing their volume and their stiffness. In addition, humidity and insufficient ventilation in the skin decrease the healing of the injury. For this reason, the therapists recommend changing their postural position during the day to decrease water accumulation and interact with the contact area with the wheelchair.

**Figure 3:**
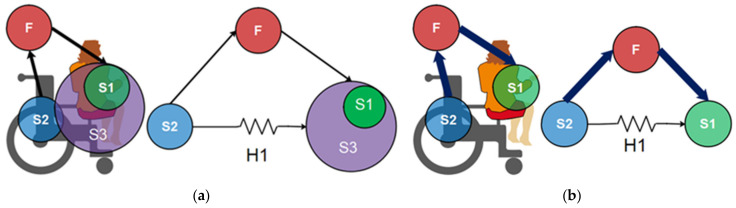
Block diagram (**a**) Su-field analysis, (**b**) Improved Su-field analysis.

For TRIZ methodology, the desired specific functions are the output from an object or substance (Si) and caused by another object or substance (Si + 1) with the help of some means (in general, it refers to types of energy, F). For example, Figure 3a represents the interaction of the wheelchair and a patient. First, the areas of contact between the patients and the wheelchair can be described as the substance (S1). Then the wheelchair represents the substance (S2), which maintains the patient in a specific postural position. In this scenario, the load applied over the contact areas (F) causes the harmful effect of ulcer formation (H1).

Thus, using the effects previously described, the device’s proposed task is to avoid ulcer skin formation in the back and gluteus. The possible function to achieve this is changing the patient’s postural position. The system has identified if the actual behavior of the system is incomplete (it means that the substance-field analysis requires completion of a new sub-system to achieve the desired effect), has an ineffective system (it requires improvement to create the desired effect), or has a complete harmful system (it requires the elimination of the negative effect). To achieve this action is used the “76 Standard Solutions” [24]. This tool of TRIZ methodology groups the viable solutions into the following five categories: standard solutions to improve the system with no or minor changes, standard solutions to improve the system by changing parts of the system, including system transitions, detect, and measuring parts of the system or implement strategies for simplification and improvement of parts in the system. In this study case, it is necessary to complete the system by adding an element between the harmful effect and the subjects. The proposed modification of the scenario is shown in Figure 3b.

This scenario applies solution 1.2.1, “Remove the harmful effects by introducing S3 substance or object”. Thus, is added a “mechanism” between the harmful effect and the skin pressure named S3. Solution 1.2.4 also points out, “The harmful effect exists in a system in which the elements S1 and S2 must be in contact. Counteract the harmful effect of F1 by having F2 neutralize the harmful effect”. Then, the “proposed mechanism” must counterbalance the payload applied over the back and the gluteus by changing the center of mass when the postural position of the patient changes from decubitus position to sit position and vice versa. In addition, if it applies to this “mechanism”, the 2.2.4 solution “divide the object or substance into parts, then make it flexible by linking the parts”. It includes flexible or movable structures between the wheelchair structure support and the reconfigurable mechanism solution to make the system more flexible.

Finally, solution 5.1.1.4, “add a super-reactive additive”, or solution 5.1.1.1 “Use nothing to add air, vacuum, bubbles, etcetera”, allows to increase the comfortability of the ventilation and decrease the humidity in the patient is a possible static solution for the cushions [25]. Table 3 summarizes the most critical parameters found through the Su-Field analysis using the contradiction matrix for solving technical parameters.

The contradiction matrix details the most critical parameters found in the analysis as follow:

- Duration of a stationary object. If the body remains too long in the same position starts to perspire. For this reason, it is decided to move parts of the body, or in this case, the upper torso of the proposed patient.
- Area of a stationary object: It is related to the division of the three parts of the wheelchair. It is decreased the area of the stationary object from three to only one.
- The object’s shape: It is related to the object’s shape to the human body’s pressure due to the low corporeal mass. As it is explained before, a quadriplegic patient’s human body is like a water balloon and oat. If the patient/balloon remains too long in the same position, the tissue’s pressure starts to swell due to the increasing water/blood on the tissue. It is also studying the correlation between pressure and perspiration on this kind of disease, but at this moment, as practical therapists have mentioned, it is always necessary to move the patient to decrease the cumulation of water (dropsy disease).

**Figure 4:**
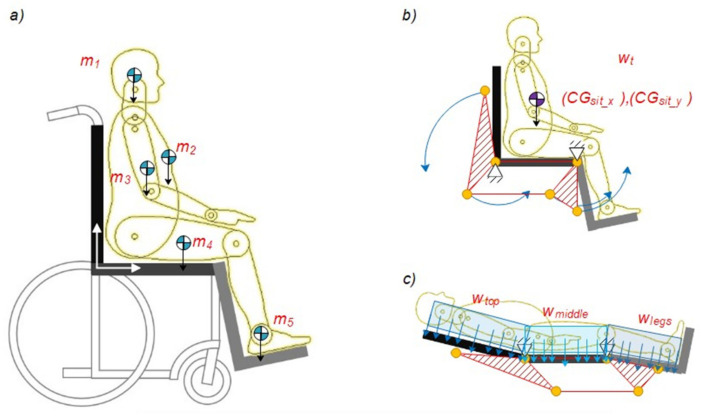
Proposed wheelchair mechanism design: (**a**) Wheelchair mainframes; (**b**) Movement of the wheelchair mechanism; (**c**) Wheelchair in the dorsal decubitus position.

Once the values are correlated, the three proposed solutions are listed next: to modify the duration of a stationary object, change the object’s shape, or modify the object’s area. By applying these solutions to a universal design of a wheelchair, Figure 4 shows the free-body diagram for the proposed solution.

Figure 4a shows the division of the wheelchair into three main parts for the subsequent analysis, the backrest, the seat, and the leg rest. In this figure, m1 represents the gravity center of the head, m2 is related to the gravity center of the chest, m3 represents the gravity center of the arms, m4 represents the computed position of the gravity center of the legs, and finally, m5 represents the center of gravity of the feet. Figure 4b shows the center of gravity CG computed when the patient is sat. Additionally, blue arrows represent the desired movement of the three main parts of the wheelchair mechanism. This free body diagram is used to calculate the dynamic analysis of the mechanism. Finally, Figure 4c represents the dummy patient’s position when it is in a decubitus position. At this stage, the static loads are defined by three uniformly distributed payloads named as W_top_ composed by the weight of the head, upper half of the torso and upper arm, the W_middle_ determined by the weight of the legs and the lower half of the torso and the W_legs_ that correspond only to legs and feet. This free body diagram is used to assess the structure of the wheelchair.

**Figure 5:**
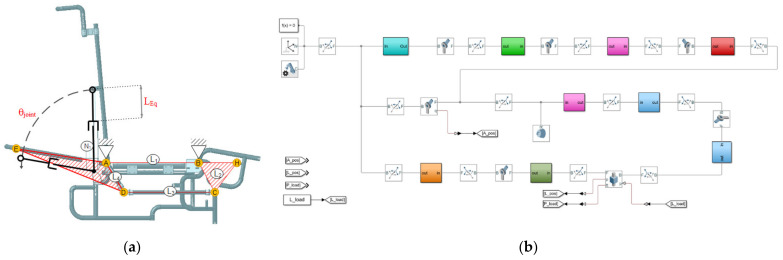
Wheelchair mechanism parts: (**a**) Wheelchair links and actuator (**b**) Wheelchair multibody diagram for position control.

Figure 5 presents the mechanism and actuator used for the mathematical plant design on the multibody analysis. Figure 5a shows the start and end positions of the mechanism and the variable of control *Leq* and the variable controlled *θjoint*. Figure 5b presents the multibody diagram of the components for the PID tuning. L1, L2, L3, and L4, represent the links of the four-bar mechanism. The linkage N1 represents the electromechanical linear actuator, Point “A” represents the system origin, Point “B” represents the joint with the chair base and the footrest mechanism, Point “C” represents the joint with the linkage conductor mechanism, Point “D” represents the reference point for the backrest, Point “E” joints the linear actuator to the backrest and the Wheelchair structure.

**Figure 6:**
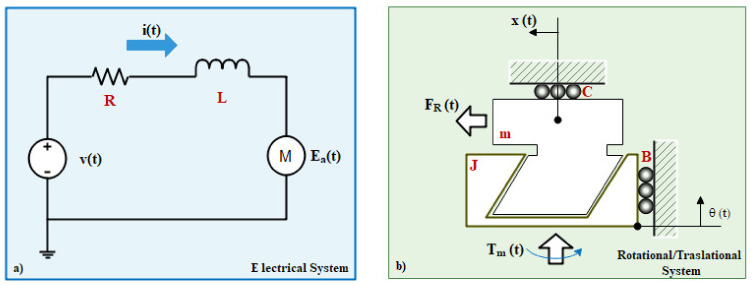
Wheelchair mechanism parts: (**a**) Idealized physical model of the DC linear motor (**b**) Screw mechanism with an interface of rotational to linear displacement.

Thus, a mathematical model of the linear actuator is proposed. The objective of this model is to relate the current consumed by the linear actuator against the extension of the rod’s actuator and then use this value to implement the proposed PID Controller. In this way, the first step is to determine the electromechanical representation of the different physical domains and correlate the elements used to model the dynamical behavior of both physical domains. The proposed schemes are shown in Figure 6. Figure 6a represents the idealized physical model of the DC linear motor. Figure 6b represents the lead screw mechanism with an interface of rotational to linear displacement.

Figure 6a presents a linear DC motor and shows the voltage of the armature through the *E_a_*(*t*) variable. Also, the internal resistance and the internal Inductance of the armature coil are represented by R and L variables. The value of the DC voltage used to energize the linear motor is v. The Current of the armature is represented by *i*(*t*). Then in Figure 6b is presented the lead screw used internally in the linear actuator to convert the motor shaft movement to the linear displacement. The usefulness of this scheme allows the conversion from rotational movement done by the motor to linear movement transferred to the lead screw-nut mechanism. In this way, the *T_m_*(*t*) represents the torque of the motor’s shaft, which applies a rotation *θ*(*t*) at *ω*(*t*) revolutions per second around the radius of the internal screw. The internal screw has the lead angle λ (commonly 14.5°) and a coefficient of friction $\mu_{s}$. This radius *r_m_* is equivalent to the pitch circle radius of the screw. Then this movement is translated to the screw-nut interface. This nut has an inertial value of J that is related to the weight of the translational shaft of the linear motor and a coefficient of rotational damping B. Then, the translational shaft has a coefficient of damping C and a mass M. This parameter is equivalent to the weight lifted by the translational shaft and is represented by *W*_eight_ divided by the earth’s gravity. This parameter is on the center of gravity CG_x_. The translational shaft *Rod_L_* has a displacement of *x*(*t*) computed by the R-RRT mechanism of the backrest *Rod_g_*. Finally, the angle of the *Rod_L_* is determined by the $\theta_{chair}$. Two dimensions give the displacement of the *Rod_L_*. The first, named *Rod_L_*, represents the static body of the linear actuator. The second one represents the shaft of the linear actuator, and it extends a distance *x*(*t*). Thus, the shaft of the linear actuator extends from the body a distance of *x*(*t*). In order to determine the numerical displacement of this parameter, the kinematic equation of the displacement was computed as:

(1) $$x\left(t\right)=-\frac{{Rod}_{L}+{Rod}_{L}\;\tan{\left(\frac{\phi }{2}\right)}^{2}-\sqrt{\sigma_{1}}}{\tan{\left(\frac{\phi }{2}\right)}^{2}+1}$$

And the angle *ϕ* is given by

(2) $$\theta_{chair}=-2\;\mathrm{atan}\left(\frac{P_{Bx}-Rod_{g}+Rod_{g}\sigma_{2}^{2}+P_{Bx}\;\sigma_{2}^{2}+\sqrt{\sigma_{1}}}{\mathrm{pBy}\sigma_{2}^{2}-2\;Rod_{g}\sigma_{2}+P_{By}}\right)$$

where:

$$\begin{array}{c} \sigma_{1}=\left(\sigma_{2}^{2}+1\right)\;\left(Rod_{g}^{2}\sigma_{2}^{2}\;+Rod_{g}^{2}+2\;Rod_{g}P_{Bx}\;\sigma_{2}^{2}-2Rod_{g}P_{Bx}-4Rod_{g}P_{By}\sigma_{2}+P_{Bx}^{2}\;\sigma_{2}^{2}+P_{Bx}^{2}\;+P_{By}^{2}\sigma_{2}^{2}+P_{By}^{2}\;\right)\\ \sigma_{2}=\tan\left(\frac{\phi }{2}\right) \end{array}$$

Then, the numerical equation that represents the dynamical behavior of the Electric domain of the DC motor is

(3) $$v\left(t\right)=E_{a}\left(t\right)+R\;i\left(t\right)+L\;i\left(t\right)$$

And the numerical equation of the torque is

(4) $$T_{m}\left(t\right)=\frac{J\;\cos\left(\lambda \right)\;\frac{\partial }{\partial t}w\left(t\right)+C\;r_{m}\;\sin\left(\lambda \right)\;\frac{\partial }{\partial t}x\left(t\right)+N\;\mu_{s}\;r_{m}\;\cos^{2}\left(\lambda \right)+N\;\mu_{s}\;r_{m}\;\sin^{2}\left(\lambda \right)-r_{m}\;F_{\mathrm{arr}}\left(t\right)\;\sin\left(\lambda \right)}{\cos\left(\lambda \right)}$$

Finally, the numerical equation that relates the displacement of the linear actuator shaft is given by

(5) $$F_{\mathrm{arr}}\left(t\right)=\frac{{\mathrm{CG}}_{x}\;W_{\mathrm{eight}}\;\cos\left(\theta_{\mathrm{ch}}\right)\;\left({Rod}_{L}+x\left(t\right)\right)}{{Rod}_{G}}$$

The constants of proportionality are listed next

(6) $$E_{a}\left(t\right)=\mathrm{Ka}\;w\left(t\right)$$

(7) $$T_{m}\left(t\right)=\mathrm{Km}\;i\left(t\right)$$

(8) $$x\left(t\right)=K_{p}\;w\left(t\right)$$

Finally, to solve Equation (3)–(8), it was applied the Laplacian operator and the Transfer function on open loop found was

(9) $$\frac{X\left(s\right)}{I\left(s\right)}=\frac{As^{2}+Bs+C\;}{Ds^{2}+Es+F}$$

where:

$A=J\;L\;{Rod}_{G}\;\cos\left(\lambda \right)+C\;K_{p}\;L\;{Rod}_{G}\;r_{m}\;\sin\left(\lambda \right)$
$B=J\;R\;{Rod}_{G}\;\cos\left(\lambda \right)+C\;K_{p}\;R\;{Rod}_{G}\;r_{m}\;\sin\left(\lambda \right)$
$C=\mathrm{Ka}\;\mathrm{Km}\;{Rod}_{G}\;\cos\left(\lambda \right)$
$D=J\;{Rod}_{G}\;\cos\left(\lambda \right)+C\;K_{p}\;{Rod}_{G}\;r_{m}\;\sin\left(\lambda \right)$
$E=-{\mathrm{CG}}_{x}\;\mathrm{Ka}\;W_{\mathrm{eight}}\;r_{m}\;\cos\left(\theta_{\mathrm{ch}}\right)\;\sin\left(\lambda \right)$
$F=-{\mathrm{CG}}_{x}\;\mathrm{Ka}\;{Rod}_{L}\;W_{\mathrm{eight}}\;r_{m}\;\cos\left(\theta_{\mathrm{ch}}\right)\;\sin\left(\lambda \right)$

Thus, it is proposed a closed-loop control system. The input for this system is given by two adjustment points, one for the stretcher position and the other for the chair position. The input can have a value ranging from 0 to 256, digitally and analogically from 0 to 5 V. The PIC 16F887 microcontroller controls the action. It evaluates the data received and generates and sends a control signal capable of modifying the position based on its algorithm. The linear actuator represents the plant, and it is the element that is to be controlled, receives the signal control, and changes gradually until reaching the desired parameters, in this case, the position. System feedback is provided by the potentiometer attached to the linear actuator. This potentiometer delivers a measurement value from 0 to 256 for the digital case or 0 to 5 V for analog. The value of this potentiometer is directly related to the position of the linear actuator.

## 4. Dynamic Analysis of the Postural Change

The geometrical and mass data of the dummy used to compute the dynamic loads are summarized in Table 4.

Equation (10) represents the general formula to assess the coordinates of the center of gravity geometrically. To determine the center of gravity of the human body, $X_{i}$ represents the ith coordinate of the ith geometrical body. The $W_{i}$ represents the weight of the ith body and $W_{t}$ represents the total weight of the body section. The same equation can be used to compute the values for $Y_{i}$ values.

(10) $$GC_{x}=\frac{\sum X_{i}W_{i}}{W_{t}};\;\;GC_{y}=\frac{\sum Y_{i}W_{i}}{W_{t}},$$

This center of gravity is used to apply the vector representing the patient’s weight over the wheelchair. By analyzing the proposed mechanism shown in Figure 4b, it can be determined the load applied by the human body to the backrest by Equation (11).

(11) $$W_{T}\;=\;\left(W_{top}+W_{middle}\right)\;=\;\left(m_{top}+m_{middle}\right)9.81\frac{m}{s^{2}},$$

By substituting the values from Table 4 in Equation (11).

$$W_{T}=608.2\;N$$

By analyzing the free-body diagram shown in Figure 5a, the force used for the linear actuator (named as N1) can be calculated as:

(12) $$\begin{array}{c} \sum M_{A}=(W_{T})\left(CG_{DdX}\right)-(F_{A})\left(0.40m\right)=0,\\ F_{A}=\frac{57.3125Nm}{0.40m}=143.3N \end{array}$$

**Figure 7:**
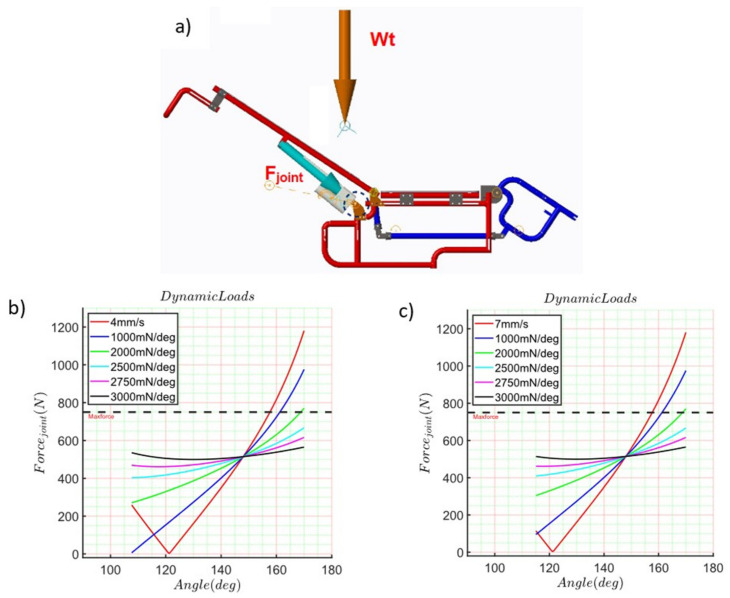
Computed results, (**a**) Dynamics wheelchair simulation; (**b**) Simulation at 4 mm/s, (**c**) Simulation at 7 mm/s.

This load was applied to the numerical simulation, as is shown in Figure 7a. The $W_{t}$ payload vector was applied on the $CG_{D}$, they are previously determined as part of the backrest parts. To make this consideration, it is done coincident the origin of the body for the different postural positions to the origin of the mechanism described as point A. On the other hand, the commercial value for the linear actuator has a maximum output of 750 N and a displacement range for the shaft of 200 mm. This value is shown in the dashed black line in Figure 7b,c. The computed results are shown in Figure 7.

Figure 7b,c represent the numerical results obtained for the load at joint A which is named as $F_{joint}$. The numerical results range from 1200 N to 250 N when the motor’s shaft is retracted at 4 mm/s and range from 1200 N to 180 N when the motor’s shaft is retracted at 7 mm/s. In both scenarios of the shaft retraction, the simulation was run for 10 s. The numerical results show that at 121° of rotation of the backrest, the load reaction increases its value until it reaches 1200 N. For this reason, It is included a torsional spring in joint A whose point of equilibrium is around 121°. The k_value_ of this torsional spring was initially proposed as a low value of 1000 mmN/deg. Then this value was increased to 500 units until it reached 3000 mmN/deg. The computed results are presented in Figure 7a for a four mm/s shaft retraction velocity and in Figure 7b for seven mm/s shaft retraction.

## 5. Structural Wheelchair Analysis

**Figure 8:**
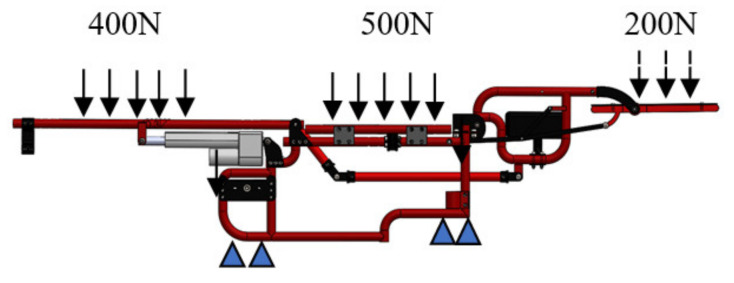
Simplify wheelchair in rest position and boundary conditions.

A CAD model of the wheelchair structure is used to evaluate the stress distribution on the four-bar mechanisms of the device. An analysis of stress and displacements that are presented in the wheelchair mechanism were performed in the Ansys Workbench^®^ software. The analyses are generated with a simplification of the system to decrease computational cost, using 197 pieces integrated by screws, tubes, blocks of polyurethane, and the electric actuators, and considering 320 points of contact and restrictions. In order to prepare the model for the numerical analysis, a three-dimensional model was imported into the ANSYS^®^ computer program, and the program correctly recognized it. The boundary conditions are shown in Figure 8.

**Figure 9:**
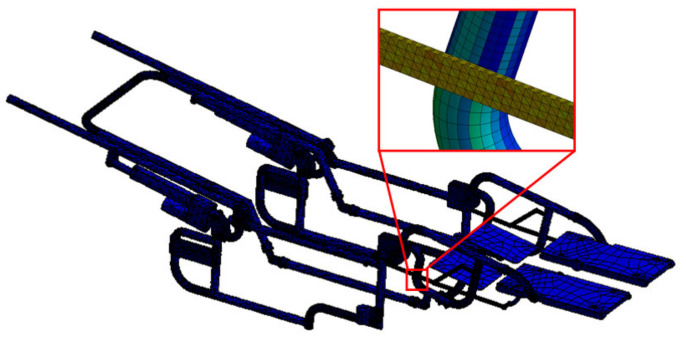
Wheelchair structure mesh.

A numerical analysis was performed by simulating the load conditions produced in the rest position due to quadriplegic human beings. Consequently, the accuracy of obtained results by numerical simulations will depend strictly on the material parameters used in their components. For example, the mechanical properties for Aluminium 6063 T-5 alloy as Young’s module of 70 GPa and the Poisson ratio of 0.33 and the mechanical properties of polypropylene was Young’s module of 1.6 GPa and the Poisson ratio of 0.42. Furthermore, the analysis was performed considering linear, elastic, and isotropic conditions. Consequently, the discretization of the three-dimensional model was done by considering tetrahedra elements. Through this procedure, the mesh consists of 880,292 nodes and 445,490 elements, Figure 9.

**Figure 10:**
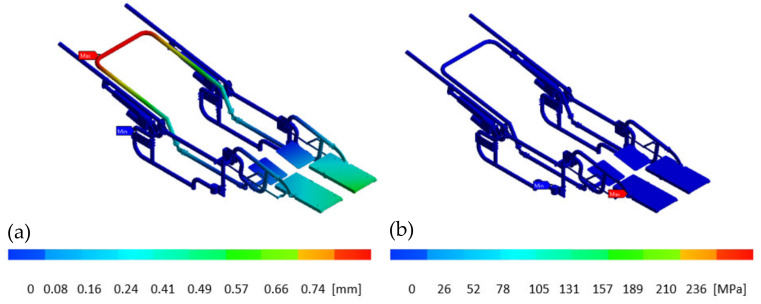
Wheelchair FEM analysis; (**a**) Deformation, (**b**) Von mises stress distribution.

Figure 10a shows the deformations on the frame and the four-bar mechanism, and Figure 10b displays Von-Misses stress distribution. These elements are subject to the most significant weight of the occupant, and it was established that the occupant’s weight is supported from the position of the axle downwards.

The computed stress values allow it to observe that the wheelchair structure works within the linear-elastic range under this load. The maximum displacements obtained by the numerical analysis of the complete system show a value of 0.74 mm. The maximum value of stress in the opposite area was 236 MPa. It should be mentioned that the allowable stress for aluminum is 280 MPa. The obtained data from the numerical analysis shows that the Von-Misses stresses produced in the wheelchair structure were lower than the elastic limit of aluminum, and the highest stress concentration is visualized under the arms movable of the footrest at the wheelchair structure. Similar values were found in [26]. It was evaluated its functional and structural aspects through simulations that consider ergonomic, anthropometric, and biomechanical factors to verify current standards.

## 6. Experimental Wheelchair Test

Finally, it was implemented a PID controller in the four-bar mechanism considering all the previous results. The main purpose of this work is to obtain a feasible and commercial device. For this reason, the plant model was computed by using MATLAB^®^ multibody toolbox. This toolbox automatically computes states’ space parameters from the functional blocks represented in the multibody system shown in Figure 5b. In addition, the numerical results were estimated for Equation (9). The linearized transfer function that describes the plant in a closed-loop for t = 3 s is shown as follow:

(13) $$F\left(s\right)=\frac{2034}{s^{2}+5496}$$

**Figure 11:**
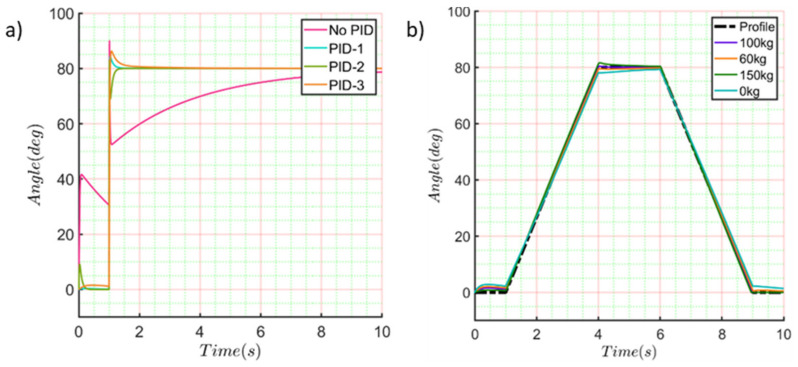
PID tunning process. (**a**) Step response for PID tuning, (**b**) Proposed trapezoidal profile for the change of position.

This transfer function was used to compute the controller PID. Next, the PID controller was tuned three times following the next procedure (Figure 11). The setpoint for all the simulations was 80 deg. First, a multibody simulation was run on the MATLAB^®^ software without loads or the PID controller block. On this point, the spring stiffness of “joint A” was not considered. The computed result is shown in Figure 11a in a pink line. After that, a block of PID controller was added and tuned using the first set of the computed results. The new response of the system is shown in Figure 11a on the blue line.

Then, joint A’s spring stiffness was set to 2.5 Nm/deg and 58 deg as an equilibrium point. The tunned PID controller block’s response is shown in Figure 11a in the green line. Finally, a punctual mass was added to link (*AE*) on the CG computed by Equation (10). The mass considered on this point was 62 kg. The computed response of this multibody simulation is shown in Figure 11a in the red line. Once the block of the PID controller was correctly tuned, a proposed trapezoidal profile for the change of position was simulated. Figure 11b shows the computed response of the entire system using the third PID block tuned values. The system’s response was evaluated by changing the punctual mass for 150 Kg, 100 Kg, and 60 Kg. The total values of each PID block tuned are summarized in Table 5.

### Controller Electronic Circuit

**Figure 12:**
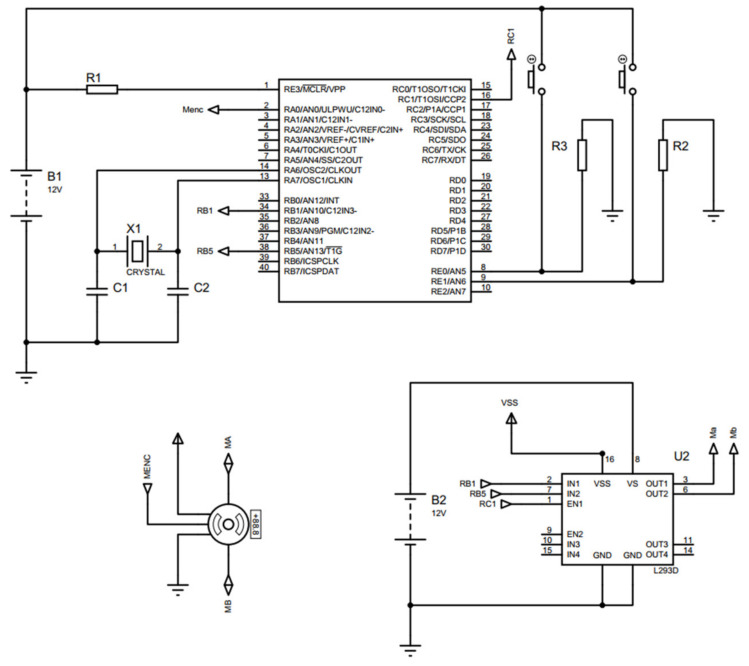
Control circuit.

The circuit shown in Figure 12 describes the control stage and the power stage used to control the wheelchair. The control stage’s main element is the microcontroller, which processes the system’s information and takes the necessary actions. Moreover, on the other hand, the power stage where the H-bridge (L293) is considered of most significant importance since it provides the necessary current for the linear actuator to develop its function.

The controller design and the electronic circuits contained in the prototype are based on the programming of the PIC16F887A microcontroller. The linear actuator implemented is of the Pololu^®^ Las Vegas, NV, USA, brand model (LACT4P-12V-20). This actuator has a gearbox with a ratio of 20:1, thanks to which it can withstand a dynamic load of up to 50 Kg (110 lb.), a maximum idle speed of 1.5 cm/s (0.59 in/s), and a maximum load speed of 1.0 cm/s (0.39 in/s). Likewise, it includes switches at the beginning and end of the race to facilitate control tasks and prevent accidents. Furthermore, it has an internal worm drive, which allows it to maintain its position when not energized.

**Figure 13:**
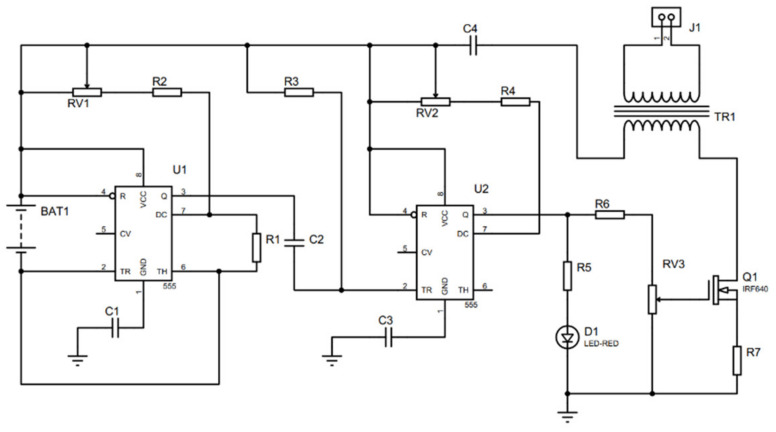
TENS Circuit.

Furthermore, this actuator has a potentiometer that has the feedback function. That is, for a particular position of the actuator, said potentiometer delivers a resistance value. The Transcutaneous Nerve Stimulator (TENS), is a system that is mainly used for the treatment of acute or chronic pain in isolation. The tissue effects are a function of the current intensity, the voltage, the frequency, the waveform, and the duration of the current passage. Depending on those variables, an analgesic and comfortable thermal effect can be obtained. The components to build the TENS circuit are shown in Figure 13.

TENS circuit was modulated between 1 mA and 120 mA of intensity, a frequency between 1 Hz to 250 Hz, and a duration of 50 to 400 μs (CNETS, 2005). Therefore, the circuit needs two 555 timer IC. The first 555 IC is configured as stable and the second as monostable. At the output, this signal is connected to a MOSFET (IRF640), to provide the necessary energy at the primary winding of the electrical power transformer. Once there, the electrical power transformer works as a voltage booster from 12 V to 120 V. The electrodes are connected. The current depends on humidity, the degree of patient’s skin, the temperature, the type of skin, the contact surface, and the contact pressure. Finally, it was selected a battery of lead-acid, from 12 V to 24.3 Ah. Thus, the performance and autonomy of the wheelchair can be assured for an average of 12 h.

**Figure 14:**
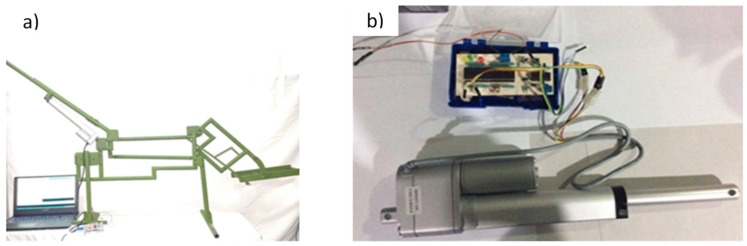
Experimental wheelchair position test; (**a**) Wheelchair prototype (**b**) Electronic circuit.

Thus, using all the previous results, an experimental test was carried out. It was manufactured with only one side of the wheelchair to test it. Figure 14a shows the prototype of the wheelchair manufactured. This part is composed of the middle of the backrest, the four-bar mechanism, and the footrest. Tubular steel was used, and the actuator was installed around joint A. Figure 14b shows the electronic circuit to control the wheelchair. Its main element is a PIC-16F887, which oversees the system’s information and implements the computed digital PID controller for the control stage. The power stage uses an H-bridge (L293), which provides the necessary current for the linear actuator.

**Figure 15:**
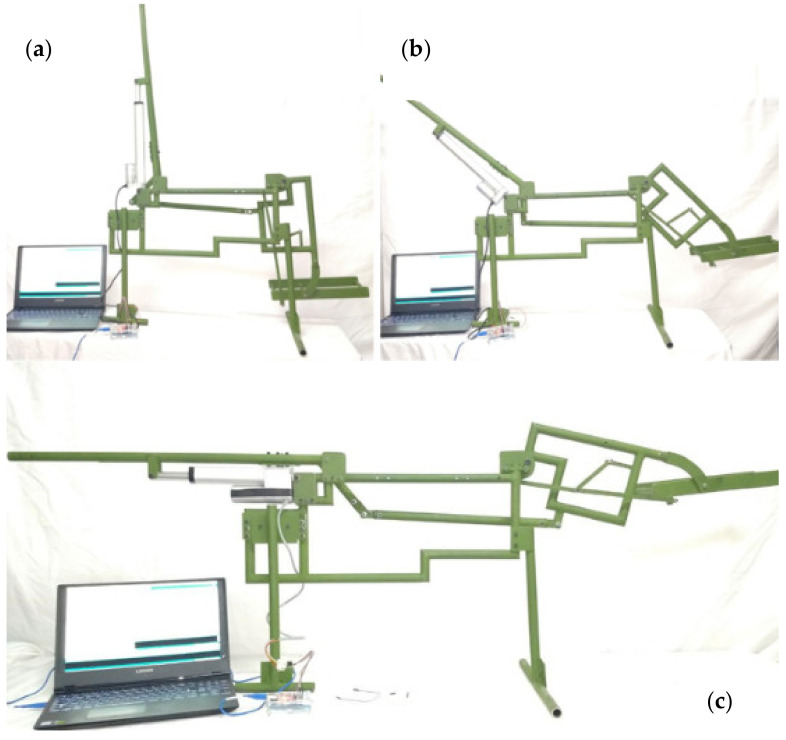
Experimental wheelchair test, (**a**) Wheelchair seat position (**b**) Wheelchair middle position (**c**) Wheelchair stretcher position.

The proposed design is presented as a novel wheelchair testbed using a lateral side as the main motion for simulation of the mechanism position. The testbed is used to give a motion and modify the patient’s orientation by different types of rests. The testbed gives a significant force and position when caregivers need to move the patients to the bed. The computed control system was applied to reach different positions within the range of motion of the wheelchair. Figure 15 shows the results of the experimental tests.

## 7. Discussions

The main contribution of this work consists of the mechanical design and easily implemented control to a wheelchair that can induce multiple changes in the postural position of patients with decreased mobility (considered as a quadriplegic patient and reduced mobility). This kind of device is used by caretakers who have limited knowledge on how to implement technological solutions to improve the quality of life in some patients. For this reason, the objective of this paper was to present some of the critical actions that untrained people implement in their daily routine to improve the quality of life of patients with decreased mobility to a completely automated device. Furthermore, those actions are used to materialize a technological product that translates behavioral actions into electromechanical tasks. Thus, an automated wheelchair controller is developed that can be implemented with minimal effort on ordinary wheelchairs by adding a four-bar mechanism. On this way, our device can be adopted quickly by untrained people and guarantee that their patient will improve their quality of life than before.

Furthermore, the wheelchair is transformed into a stretcher by integrating a four bars mechanism into the original support structure. This solution was achieved by implementing the TRIZ methodology and the substance field Tool to determine possible general solutions. By implementing these solutions, it is decreased the probability of ulcer formation. Also, If the patient has dropsy disease, it is moved continuously in their limbs. The main advantage of this design is the patient’s load distribution; for this case, more than 50% is supported by the chair’s sitting, and the mobile mass corresponds to more than half of the patient’s length. However, this solution was achieved under certain limits. The first of them is the proposed mechanism. This mechanism shows that the payload critic is around 500 N at 148.3 deg. To decrease this value, it is implementing a torsional spring in joint A that helps to decrease the momentum around this joint. In the beginning, it is not known how much stiffness it must have in the spring.

For this reason, it is started to run different simulations from 1000 mmN/deg and increase the proposed value until the payload had changed the computed slope for the load reaction. Finally, this value was reached at 2500 mmN/deg. With this value, it should reach different angles of the backrest and the footrest of the wheelchair by using the same linear actuator and maintaining the mechanism’s position without reaching 100% of the maximum force exerted by the linear actuator. Once it is found the new parameters of the system, It is also exported the dynamical loads computed for the new conditions. This was done to verify if the mechanism supports the payload caused by the new dynamical situation. Fortunately, the numerical simulations show that joint A has an average maximum stress of 150 MPa, representing 63% of the maximum allowable stress of the material. Therefore, in future versions of the mechanism’s manufacture, it is going to change the material of this part to decrease these values and have a more conservative value for non-considered conditions. Similarly, in the numerical simulation of the dynamical conditions, it is found that the mechanism is deflected by 0.4 mm. This value is not high enough to increase friction between mobile parts and ensure that this parameter is included as a minimal tolerance requirement for the parts separation during manufacture.

**Figure 16:**
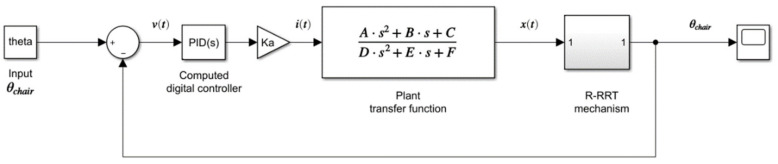
Experimental wheelchair position test.

Lastly, one of the main goals is to achieve a mechanism that is easy to implement in actual wheelchairs, capable of controlling the mechanism using an electronic PID controller shown in Figure 16. For this reason, it is decided to linearize the plant model by using the MATLAB^®^ toolbox.

Thus, a novel wheelchair posture device is presented to highlight the influence of Mechanism Design in modern systems [27]. However, it is essential to mention that this design does not contemplate using a headrest [28]. Therefore, it cannot be used as a permanent stretcher [29]. Furthermore, the degree of automation proposed in this design does not allow it to be an electric wheelchair; only are established the control circuits necessary to achieve the device’s multiposition [30].

## 8. Conclusions

In this work, is applied TRIZ methodology to propose a dynamic multiposition wheelchair prototype. The main characteristics reported in similar designs were summarized. Evidence from this study has suggested that the main problem for novel designs should be focused on preventing ulcer formation in patients by changing the static object area. It is applied the methodology of the 76 standard solutions. It was proposed to solve these problems by making the system more flexible and changing its surface contact with the skin. It is proposed to use a four-bar mechanism that changes the structure of a standard wheelchair. This structure changes, allowing multiple positions for common postural to be adopted. The sitting position and the decubitus position. The whole mechanism uses an electromechanical linear actuator controlled by a PID control. The controllers’ design consisted of calculating the variables of the PID controller.

## Figures and Tables

**Table 1:** Wheelchair components description.

Reference	Component	Description
A	Rear-wheel	600 mm diameter (standard market size)
B	Front-wheel	200 mm diameter. (standard market size)
C	Armrests	Adjustable armrests with 90° position of the arm about the forearm.
D	Battery Base	It is placed under the seat, with dimensions for battery support.
E	Backrest	Its design is based on the dimensions obtained from the synthesis of the four-bar mechanism based on the TRIZ methodology, allowing the stabilization of the occupant’s upper lumbar region.
F	Seat	The objective is to stabilize the occupant’s upper lumbar region.
G	Leg rest	Support for the calf and foot.
H	Handle	It is made of a single piece joined to the back that forms the structure of the chair
I	Brakes	Brakes leading shoe mounted on the tube under the seat and activated by pushing forward.
J	Posture Control.	Designed to establish the occupant’s desired posture, easy to operate
K	TENS	Transcutaneous Nerve Stimulation System, which provides analgesic treatment, with power indicator and two modulating knobs

**Table 2:** Main parameters for wheelchair design.

Dimensional Parameters	Functional Parameters
The weight of the people is around 100 kg	Position movements between sit and dorsal decubitus.
The human body is divided into five parts: head, chest, upper limbs, lower limbs, and foot	One day of minimal time of autonomy
The horizontal position of the backrest is reached at 170°	Proposal for a friendly user interface to control the movement of the entire device
The backrest dimension is 70 cm, the length of the seat is 45 cm, and the length of the footrest is 16 cm	Use of low-cost materials to design the electronic interfaces
The material of the wheelchair structure is Aluminum 6063 T5	Proposal of electronic device for lower focalized pain
Power consumption less than 30,000 mA-h	Maximum exploitation of actual structure of ordinary wheelchairs to insert the proposed mechanism

**Table 3:** Main design parameters for the proposed dynamic wheelchair.

	40 Technical Troubles to Be Solved				
39 Technical parameters	2.-Reduce the temperature	6.-To move an object	12.-To generate or to control payloads	13.-To change the friction between two objects	Total
2.-Weight of a stationary object	----	X	X	----	2
6.-Area of a stationary object	X	X	X	X	4
10.-Force (intensity)	----	----	X	----	1
11.-Stress or pressure	----	----	X	----	1
12.-Shape	X	X	X	X	4
14.-Strength	----	X	X	----	2
16.-Duration of action by a stationary object	X	X	X	X	4
17.-Temperature	X	----	----	X	2
31.-Object-generated harmful factors	X	----	----	X	2
33.-Convince of use	----	X	----	----	1

**Table 4:** Geometrical values of body parts.

Body Part	Approximated Weight (Kg)	Sitting Position		Decubitus Dorsal	
		X (mm)	Y (mm)	X (mm)	Y (mm)
Head	7	10	62	−77.5	10
Chest	44	10	45	−38	10
Arms	11	25	20	−45.28	6
Legs	34.5	50	−17	23.09	6
Foot	3.5	62.5	−35.50	85.50	16

**Table 5:** Computed values for the tunning process of PID controller.

Variable	PID-1st Tunning	PID-2nd Tunning	PID-3rd Tunning
Proportional (P)	0.93257	10.8561	41.1214
Integrative (I)	1.1769	162.9655	23.1349
Derivative (D)	0.08092	0.17607	6.7003
Filter coefficient (N)	18,841.0383	42,806.4155	8183.4159
Rising time	1.12 × 10^−2^ s	4.58 × 10^−3^ s	2.51 × 10^−2^ s
Settling point	1.66 × 10^−1^ s	1.182 × 10^−1^ s	2.88 × 10^−1^ s
Overshoot	5.64%	3.78%	6.43%
Peak	1.06	1.04	1.06

## Data Availability

Not applicable.

## References

[1] Routier F., Gagnon B., Lemelin B. (2015). Effect of wheelchair tires types and weight on wheelchair propulsion. Ann. Phys. Rehabil. Med..

[2] Sarraj A.R., Massarelli R. (2011). Design history and advantages of a new lever-propelled wheelchair prototype. Int. J. Adv. Robot. Syst..

[3] Rice I. (2016). Recent Salient Literature Pertaining to the Use of Technology in Wheelchair Sports. Curr. Phys. Med. Rehabil. Rep..

[4] Kloosterman M.G.M., Eising H., Schaake L., Buurke J.H., Rietman J.S. (2011). Comparison of shoulder load during power-assisted and purely hand-rim wheelchair propulsion. Clin. Biomech. (Bristol Avon).

[5] Mori Y., Sakai N., Katsamura K. (2012). Development of a Wheelchair with a Lifting Function. Adv. Mech. Eng..

[6] Chow J.W., Levy C.E. (2011). Wheelchair propulsion biomechanics and wheelers’ quality of life: An exploratory review. Disabil. Rehabil. Assist. Technol..

[7] Quaglia G., Bonisoli E., Cavallone P. (2018). A proposal of alternative propulsion system for manual wheelchair. Int. J. Mech. Control..

[8] Huang G., Zhang W., Yu Z., Chen X., Meng F., Ceccarelli M., Huang Q. (2017). Design and simulation of leg exoskeleton cycling-actuated wheelchair. Int. J. Adv. Robot. Syst..

[9] Gagnon D., Roy A., Gabison S., Duclos C., Verrier M.C., Nadeau S. (2016). Effects of Seated Postural Stability and Trunk and Upper Extremity Strength on Performance during Manual Wheelchair Propulsion Tests in Individuals with Spinal Cord Injury: An Exploratory Study. Rehabil. Res. Pract..

[10] Ning M., Yu K., Zhang C., Wu Z., Wang Y. (2021). Wheelchair design with variable posture adjustment and obstacle-overcoming ability. J. Braz. Soc. Mech. Sci. Eng..

[11] Hng Lim S., Kiat Ng P. (2021). The Design and Development of a Foldable Wheelchair Stretcher. Inventions.

[12] Xi Z., Meng-Di Y. (2021). Research on Wheelchair Design for the Disabled Elderly Based on QFD/TRIZ. J. Phys. Conf. Ser..

[13] Shi X., Lu H., Chen Z. (2021). Design and Analysis of an Intelligent Toilet Wheelchair Based on Planar 2 DOF Parallel Mechanism with Coupling Branch Chains. Sensors.

[14] Shaikh-Mohammed J., Sourav Dash S., Sarda V., Sujatha S. (2021). Design journey of an affordable manual standing wheelchair. Disabil. Rehabil. Assist. Technol..

[15] Murray R., Sunil K.C., Ophaswongse A. Design of Wheelchair Robot for Active Postural Support (WRAPS) for Users with Trunk Impairments. Proceedings of the ASME 2018 International Design Engineering.

[16] Quaglia G., Bonisoli E., Cavallone P. (2019). The Design of a New Manual Wheelchair for Sport. Machines.

[17] Gumasing M.J., Villapando A.C., Pernia K.C. (2019). An Ergonomic Design of Wheelchair Bed Transfer for Post Stroke Patients. Proceedings of the 2019 International Conference on Management Science and Industrial Engineering.

[18] Alkhateeb A.M., Daher N.S., Forrester B., Martin B.D., Jaber H.M. (2019). Effects of adjustments to wheelchair seat to back support angle on head, neck, and shoulder postures in subjects with cerebral palsy. Assist. Technol. Off. J. RESNA.

[19] Hong E., Dicianno B.E., Pearlman J., Cooper R. (2014). Comfort and stability of wheelchair backrests according to the TAWC (tool for assessing wheelchair discomfort). Disabil. Rehabil. Assist. Technol..

[20] Murray R., Ophaswongse C., Agrawal S.K. Design of a Wheelchair Robot for Active Postural Support. Proceedings of the ASME 2018 International Design Engineering Technical Conferences and Computers and Information in Engineering Conference.

[21] Shirogane S., Takashi H., Kozai Y., Maeda Y. (2017). A preliminary study of the measurement of overload applied to the foot support of a wheelchair and a seated postural support device. J. Phys. Ther. Sci..

[22] Crytzer T., Hong E., Dicianno B.E., Pearlman J., Schmeler M., Cooper R.A. (2016). Identifying characteristic back shapes from anatomical scans of wheelchair users to improve seating design. Med. Eng. Phys..

[23] Tessari R.K., De Carvalho M.A. (2015). Compilation of Heuristics for Inventive Problem Solving. Procedia Eng..

[24] Altshuller G.S. (1989). The Art of Inventing and Suddenly the Inventor Appeared.

[25] Russo D., Duci S. (2015). From Altshuller’s 76 Standard Solutions to a New Set of 111 Standards. Procedia Eng..

[26] Santos Marques L., Rodrigues Magalhaes R., de Lima D.A., Esquina Tsuchida J., Cardoso Fuzzato D., de Andrade E.T. (2020). Finite element analysis of a commercial wheelchair. Disabil. Rehabil. Assist. Technol..

[27] Desai S., Mantha S., Phalle V. (2019). TRIZ and AHP in Early Design Stage of a Novel Reconfigurable Wheelchair. J. Mech. Eng..

[28] Gao H., Tang Y., Wan Z., Wu Q. (2021). Design of a multifuntional nursing wheelchair. J. Phys. Conf. Ser..

[29] Baskar C., Anitha N., Arun Kumar S., Deepak Kumar D. (2021). Integrated bed cum wheelchair system for crippled patients. Mater. Today Proc..

[30] Ahmed F., Paul R., Ahmad M.M., Ahammad A., Singha S. Design and Development of a Smart Wheelchair for the Disabled People. Proceedings of the IEEE International Conference on Information and Communication Technology for Sustainable Development (ICICT4SD).

